# Immune cells promote paralytic disease in mice infected with enterovirus D68

**DOI:** 10.1172/JCI188495

**Published:** 2025-06-03

**Authors:** Mikal A. Woods Acevedo, Lan, Sarah Maya, Jennifer E. Jones, Isabella E. Bosco, John V. Williams, Megan Culler Freeman, Terence S. Dermody

**Affiliations:** 1 Department of Pediatrics, University of Pittsburgh School of Medicine, Pittsburgh, Pennsylvania, USA.; 2 Institute of Infection, Inflammation, and Immunity, UPMC Children’s Hospital of Pittsburgh, Pittsburgh, Pennsylvania, USA.; 3 Department of Microbiology and Molecular Genetics, University of Pittsburgh School of Medicine, Pittsburgh, Pennsylvania, USA.

**Keywords:** Immunology, Infectious disease, Virology, Cellular immune response, T cells

## Abstract

Enterovirus D68 (EV-D68) is associated with acute flaccid myelitis (AFM), a poliomyelitis-like illness causing paralysis in young children. However, the mechanisms of paralysis are unclear, and antiviral therapies are lacking. To better understand EV-D68 disease, we inoculated newborn mice intracranially to assess viral tropism, virulence, and immune responses. WT mice inoculated intracranially with a neurovirulent strain of EV-D68 showed infection of spinal cord neurons and developed paralysis. Spinal tissue from infected mice revealed increased levels of chemokines, inflammatory monocytes, macrophages, and T cells relative to those in controls, suggesting that immune cell infiltration influences pathogenesis. To define the contribution of cytokine-mediated immune cell recruitment to disease, we inoculated mice lacking CCR2, a receptor for several EV-D68–upregulated cytokines, or RAG1, which is required for lymphocyte maturation. WT, *Ccr2^–/–^*, and *Rag1^–/–^* mice had comparable viral titers in spinal tissue. However, *Ccr2^–/–^* and *Rag1^–/–^* mice were significantly less likely to be paralyzed relative to WT mice. Consistent with impaired T cell recruitment to sites of infection in *Ccr2^–/–^* and *Rag1^–/–^* mice, antibody-mediated depletion of CD4^+^ or CD8^+^ T cells from WT mice diminished paralysis. These results indicate that immune cell recruitment to the spinal cord promotes EV-D68–associated paralysis and illuminate potential new targets for therapeutic intervention.

## Abstract 2

EV-D68 is a pathogen associated with a poliomyelitis-like illness in young children. Immune cell recruitment to the spinal cord promotes EV-D68 paralysis in newborn mice.

## Graphical abstract

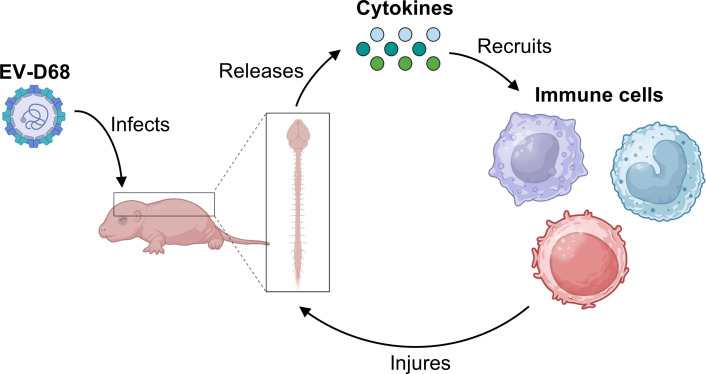

## Introduction

Enteroviruses cause a wide spectrum of disease in humans, including acute flaccid myelitis (AFM), a poliomyelitis-like paralytic condition that occurs primarily in children (1, 2). AFM is thought to result from injury to spinal cord motor neurons (3, 4), although the mechanisms of cell killing are unclear. Enterovirus D68 (EV-D68) was first detected in children with pneumonia in 1962 (5) and is considered a reemergent pathogen after its association with AFM. The CDC began tracking AFM outbreaks in 2014 (6, 7), but EV-D68 has been associated with paralysis as early as 2008 (3, 8). There are currently no targeted treatments for EV-D68 infection or AFM (9). Therefore, there is an urgent need to define mechanisms by which EV-D68 causes disease.

Other neurotropic viruses, such as poliovirus, cause limb paralysis by inducing apoptosis in spinal cord motor neurons (10). In human spinal cord organoids (hSCOs) lacking immune cells, EV-D68 replicates but causes minimal cell death relative to other enteroviruses (11), suggesting that EV-D68 replication is not the sole mediator of neuronal cell death. Cerebral spinal fluid (CSF) obtained from persons with AFM rarely contains evidence of a pathogen (12), but is often enriched for enterovirus-specific antibodies (13, 14). Postmortem studies of a child with flaccid paralysis yielded EV-D68 RNA in the CSF and identified EV-D68 capsid protein and RNA, CD68^+^ macrophages, and CD8^+^ T cells in the spinal cord (3, 8). Furthermore, spinal cord sections stained negative for caspase-3 but positive for perforin, suggesting that the child’s immune response, and not virus-induced apoptosis, contributed to the disease (3). Immune cells can exacerbate certain virus-induced diseases. In mice, CD8^+^ T cells contribute to Zika virus–associated paralysis (15) and lymphocytic choriomeningitis virus–related mortality (16). Mature lymphocytes are implicated in damaging myelin after spinal cord injury, which restricts recovery (17). Uncovering the mechanisms by which EV-D68 and the subsequent immune response contribute to disease progression could potentially lead to the identification of strategies for therapeutic intervention.

Several in vitro and ex vivo models are available to study EV-D68 neural infection and pathogenesis (18). Contemporary neurotropic EV-D68 strains, but not historical non-neurotropic strains, efficiently replicate in human neuronal cell lines (19), with replication efficiency in human neuroblastoma cells attributable to sequence polymorphisms in viral capsid proteins (20). In murine organotypic brain slice cultures, EV-D68 infects Nissl-stained neurons (21). In primary rat cortical neurons, EV-D68 infects both excitatory glutamatergic and inhibitory GABAergic neurons (22). Cultivated human B cells and dendritic cells, but not CD4^+^ or CD8^+^ T cells, stain positive for EV-D68 capsid protein (23), suggesting that both neurons and certain immune cell subsets can be infected by EV-D68 in humans. However, while these models allow studies of EV-D68 infection of cells, they do not recapitulate EV-D68 disease in a complex host environment.

Animal models of EV-D68 disease reproduce important aspects of infection and disease in humans (18). In newborn mice, EV-D68 infection causes an AFM-like illness (9) to which mice become resistant as they age (24). Additionally, the inoculation route and viral dose dictate the efficiency with which EV-D68 causes paralysis, with intracranial inoculations being among the most neuropathogenic routes (9, 18). In mice inoculated intracranially, one-tenth the dose of EV-D68 is required to achieve similar viral loads in the spinal cord and paralysis onset relative to those inoculated intraperitoneally (25). Thus, neonatal mice inoculated intracranially serve as useful experimental models to study mechanisms by which EV-D68 causes AFM.

In this study, we examined the effect of host immunity on EV-D68 replication in the central nervous system (CNS) and how it influences disease development in newborn mice. We observed an EV-D68 strain–specific cytokine response in both hSCOs and newborn mice, which had robust immune cell recruitment to the spinal cord. Additionally, we observed that mice lacking functional immune cell recruitment or mature lymphocytes had diminished disease relative to immunocompetent mice. Antibody-mediated depletion of CD4^+^ or CD8^+^ T cells resulted in substantial protection against EV-D68–associated AFM relative to isotype control antibody. Collectively, these findings suggest that immune cells recruited to the spinal cord promote development of EV-D68–associated AFM.

## Results

### EV-D68 strains differ in virulence and immune responses in immunocompetent neonatal WT mice.

**Figure 1:**
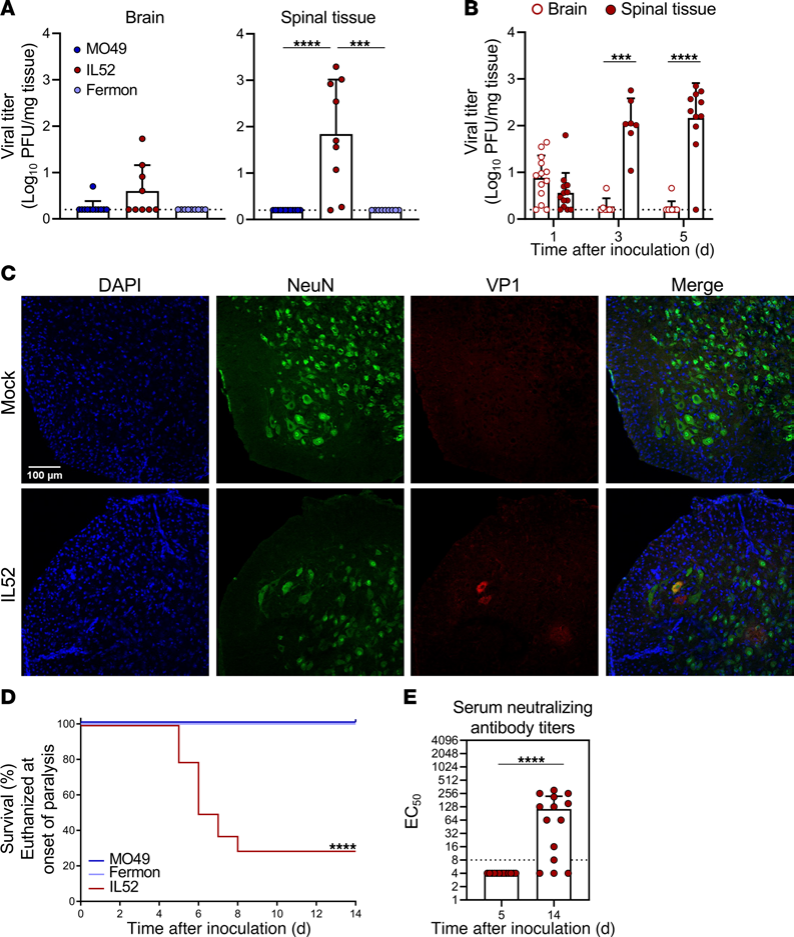
EV-D68 US/IL/14-18952 infects and causes paralysis in immunocompetent neonatal mice. Three-day-old WT mice were inoculated i.c. with PBS (mock), 10^5^ PFU EV-D68 USA/Fermon (Fermon), US/MO/14-18949 (MO49), or US/IL/14-18952 (IL52). (**A**) Brain and spinal tissue were resected at 3 dpi from EV-D68–inoculated mice, and viral titers were determined by plaque assay. (**B**) Brain and spinal tissue were resected from IL52-inoculated mice at 1, 3, or 5 dpi, and viral titers were determined by plaque assay. (**C**) Spinal columns were resected at 5 dpi from IL52-inoculated paralyzed mice or day-matched mock-inoculated controls. Samples were processed for immunohistochemistry staining. DAPI, blue; NeuN, green; EV-D68 VP1, red. Scale bar: 100 μM. (**D**) Inoculated mice were monitored daily and euthanized upon signs of paralysis. *n* = 12–27 mice per group. (**E**) Neutralizing antibody titers in sera collected at 5 or 14 dpi from IL52-inoculated mice. Dotted lines indicate the limit of detection. Data are representative of 2–3 independent experiments. Each symbol represents an individual mouse. Error bars indicate mean ± SD. Mann-Whitney *U* test (**A**, **B**, and **E**) or log-rank test (**D**): ***P ≤ 0.001; ****P ≤ 0.0001.

To understand how EV-D68 causes paralysis, we recovered the prototype USA/Fermon strain, non-mouse-adapted US/MO/14-18949 (MO49) (25), and virulent strain US/IL/14-18952 (IL52) (25) from cDNA clones. We inoculated 3-day-old C57BL/6J WT mice intracranially with 10^5^ PFU of each strain. At 3 days after inoculation (dpi), we resected brains and spinal columns from infected mice and determined viral loads in homogenized tissues by plaque assay (Figure 1A). Only mice inoculated with EV-D68 IL52 had appreciable viral titers in spinal tissue, which prompted us to examine the viral replication kinetics of this strain in brain and spinal tissue by determining titers at 1, 3, and 5 dpi (Figure 1B). Viral loads at the site of inoculation were detectable predominantly at 1 dpi, whereas viral loads in the spinal column increased over time. To define sites of EV-D68 infection in the spinal column, we stained spinal cord sections for the EV-D68 VP1 capsid protein and observed viral antigen associated with NeuN-marked neurons in infected animals (Figure 1C). Additionally, EV-D68 IL52 was the only strain tested that caused paralysis in WT mice (Figure 1D), as has been reported previously (9, 25). However, all EV-D68 strains tested replicated in human BEAS-2B lung epithelial cells (Supplemental Figure 1, A–C; supplemental material available online with this article; https://doi.org/10.1172/JCI188495DS1). In contrast to AFM in humans (26), there was no observable pattern in the distribution of limbs affected by paralysis in infected mice (Supplemental Figure 2A). There were no detectable neutralizing antibodies in the serum at 5 dpi, which included 5 mice with and 8 mice without paralysis (Figure 1E). However, 11 of 35 mice inoculated with EV-D68 IL52 that did not develop paralysis by 14 dpi had detectable EV-D68–neutralizing antibodies (Figure 1E), indicating that paralysis following infection was not universal. Collectively, these results demonstrate that some strains of EV-D68 replicate, cause disease, and elicit neutralizing antibodies in neonatal WT mice inoculated intracranially.

### EV-D68 strains differ in cytokine responses in spinal tissue of WT mice.

**Figure 2:**
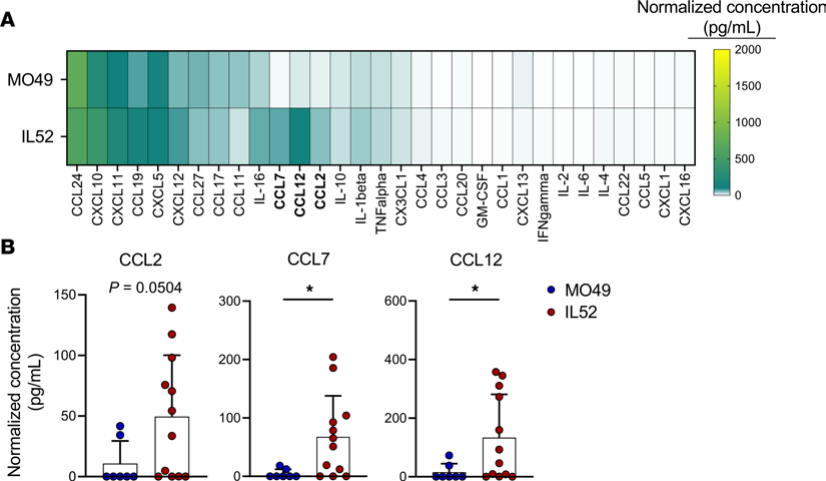
Cytokines in spinal tissue of EV-D68–inoculated mice. Three-day-old WT mice were inoculated i.c. with MO49, IL52, or PBS (mock). Spinal tissue was resected 3 dpi and analyzed by Luminex protein assay. (**A**) Cytokine concentrations are presented as mean values in pg/mL from 7–12 mice and shown as a heatmap normalized to concentrations in mock-inoculated mice. (**B**) CCL2, CCL7, and CCL12 concentrations normalized to those in mock-inoculated mice are shown. Data are representative of 2–3 independent experiments. Each symbol represents an individual mouse. Error bars indicate mean ± SD. Mann-Whitney *U* test (**B**): *P ≤ 0.05.

To characterize the host response to EV-D68, we conducted multianalyte Luminex-based profiling of 31 proinflammatory cytokines in spinal tissue of WT mice that were inoculated with PBS as a control or the EV-D68 strains MO49 or IL52 (Figure 2A). At 3 dpi, a subset of cytokines was elevated in spinal tissue from mice inoculated with either virus strain. However, mice inoculated with the neurovirulent IL52 strain had significantly higher levels of CCL7 and CCL12 and nonsignificantly higher levels of CCL2 relative to mice inoculated with EV-D68 MO49 (Figure 2B). These results indicate that a neurovirulent EV-D68 strain elicits a robust cytokine response in spinal tissue of WT mice.

### Neurovirulent EV-D68 infects hSCOs and elicits a cytokine response that mimics the murine cytokine response.

**Figure 3:**
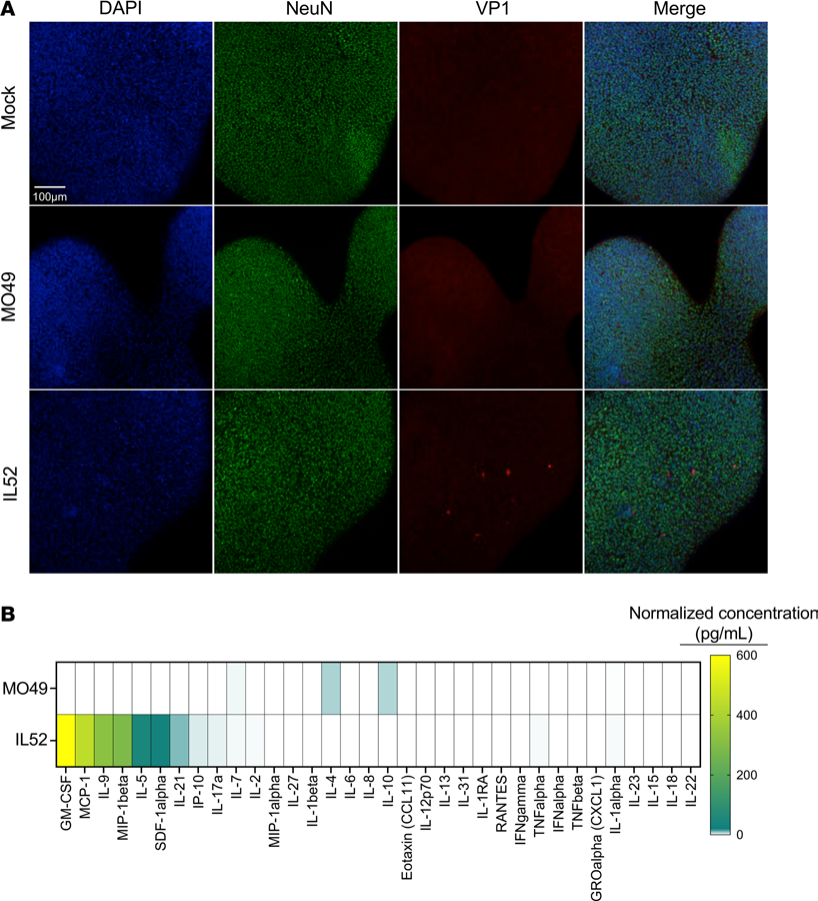
Neurovirulent EV-D68 efficiently infects hSCOs. Pools of 8–12 hSCOs at 14 days after differentiation were either mock inoculated (PBS) or inoculated with 10^5^ PFU EV-D68 MO49 or IL52. (**A**) At 3 dpi, whole organoids were processed for immunofluorescence. DAPI, blue; NeuN, green; EV-D68 VP1, red. (**B**) hSCO supernatants were collected at 3 dpi and analyzed by Luminex protein assay. Protein levels are presented as mean values in pg/mL from 2–3 independent samples and shown as a heatmap normalized to levels in mock-infected organoids.

It is unclear which cells contribute to the different cytokine responses elicited in neonatal mice by different EV-D68 strains. To determine how a multicellular organoid responds to viral infection in the absence of immune cells and to assess whether the cytokine response in this system mimics that observed in mice, we used a human-derived 3-dimensional spinal cord organoid (3-DiSC hSCO) system. We inoculated pools of 8–12 organoids with PBS (mock) or with EV-D68 strains MO49 or IL52 and monitored infection and cytokine levels. Consistent with the viral load trends in murine neural tissues, we observed VP1 staining in organoids inoculated with IL52, but not MO49, at 3 dpi (Figure 3A). We next examined virus-mediated cytokine profiles in 3DiSC hSCO supernatants harvested at 3 dpi (Figure 3B). Inoculation with EV-D68 IL52 resulted in cytokine induction similar to that in mice. For example, MCP-1, also known as CCL2, was elevated in both EV-D68 IL52–inoculated 3-DiSC hSCOs and in mice. The mean normalized concentration of MCP-1 was 21 pg/mL for MO49 and 443 pg/mL for IL52. Together, these results indicate that neurovirulent EV-D68 IL52 infected 3-DiSC hSCOs and induced a cytokine profile comparable to that in murine spinal tissue.

### Spinal cords of paralyzed mice have increased immune cell populations.

**Figure 4:**
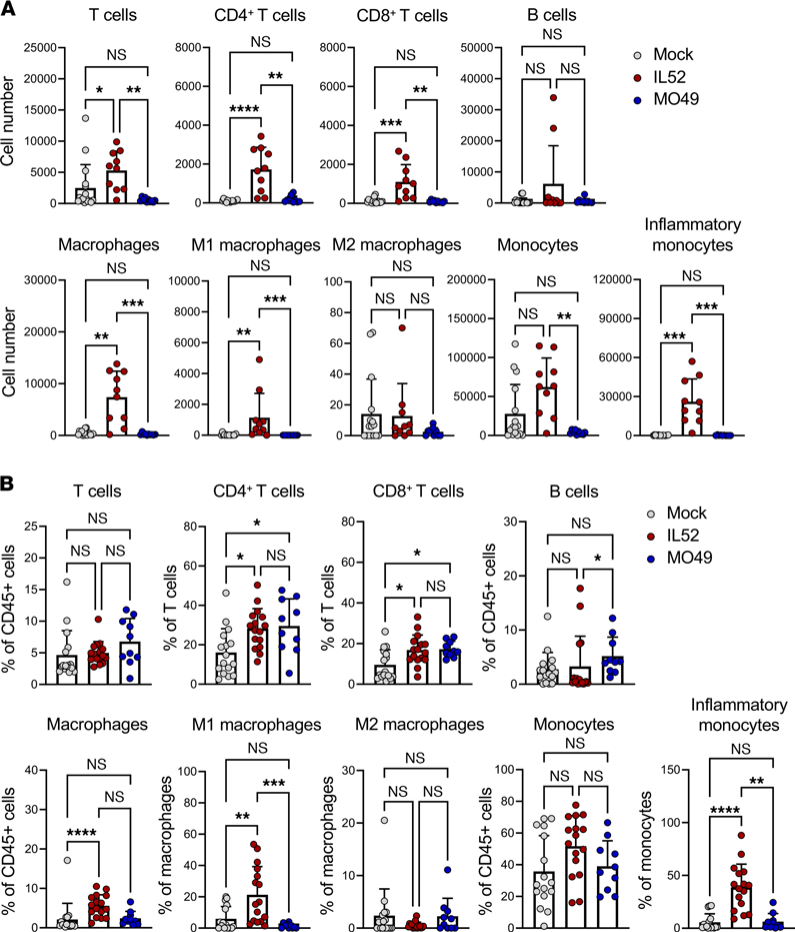
Mice inoculated with neurovirulent EV-D68 have altered populations of immune cells in the spinal cord. Three-day-old WT mice were inoculated i.c. with PBS (mock), EV-D68 MO49, or IL52. Spinal cords were resected from paralyzed IL52-inoculated mice or day-matched mice inoculated with MO49 or PBS (mock). Single-cell suspensions were prepared, stained, and analyzed by flow cytometry. (**A**) Numbers and (**B**) percentages of the indicated cell types are shown. Data are representative of 2–4 independent experiments. Each symbol represents an individual mouse. Error bars indicate mean ± SD. Kruskal-Wallis test: *P ≤ 0.05; **P ≤ 0.01; ***P ≤ 0.001; ****P ≤ 0.0001.

Since spinal tissue of mice inoculated with EV-D68 IL52 had increased levels of chemoattractant cytokines relative to mice inoculated with PBS or EV-D68 MO49, we hypothesized that neurovirulent EV-D68 infection leads to immune cell recruitment to the spinal cord. Furthermore, CD8^+^ T cells and CD68^+^ macrophages were evident in spinal cord sections containing enterovirus antigen from a child who died of AFM (8), suggesting that cellular immunity contributes to AFM pathogenesis. To assess immune cells in the spinal cord of paralyzed mice, we examined spinal cord single-cell suspensions using a multiplex 27-parameter flow cytometry panel (Supplemental Table 1 and Supplemental Figure 3). Spinal cords were resected from mice inoculated with EV-D68 IL52 that displayed signs of paralysis or day-matched controls inoculated with PBS or EV-D68 MO49 and analyzed by flow cytometry (Figure 4A). There were significant increases in the numbers of total T cells, CD4^+^ T cells, CD8^+^ T cells, total macrophages, M1 macrophages, total monocytes, and inflammatory monocytes in the spinal cord of EV-D68 IL52–inoculated mice relative to those inoculated with PBS or EV-D68 MO49. There also was an increased percentage of CD4^+^ T cells, CD8^+^ T cells, total macrophages, M1 macrophages, and inflammatory monocytes in the spinal cord of EV-D68 IL52–inoculated mice relative to controls (Figure 4B). Levels of other immune cell populations varied modestly or did not differ significantly between groups (Supplemental Figure 4, A and B). These results suggest that several types of innate and adaptive immune cells are increased in the spinal cord of paralyzed mice following inoculation with IL52.

### CCR2-dependent immune cell recruitment influences paralysis frequency.

**Figure 5:**
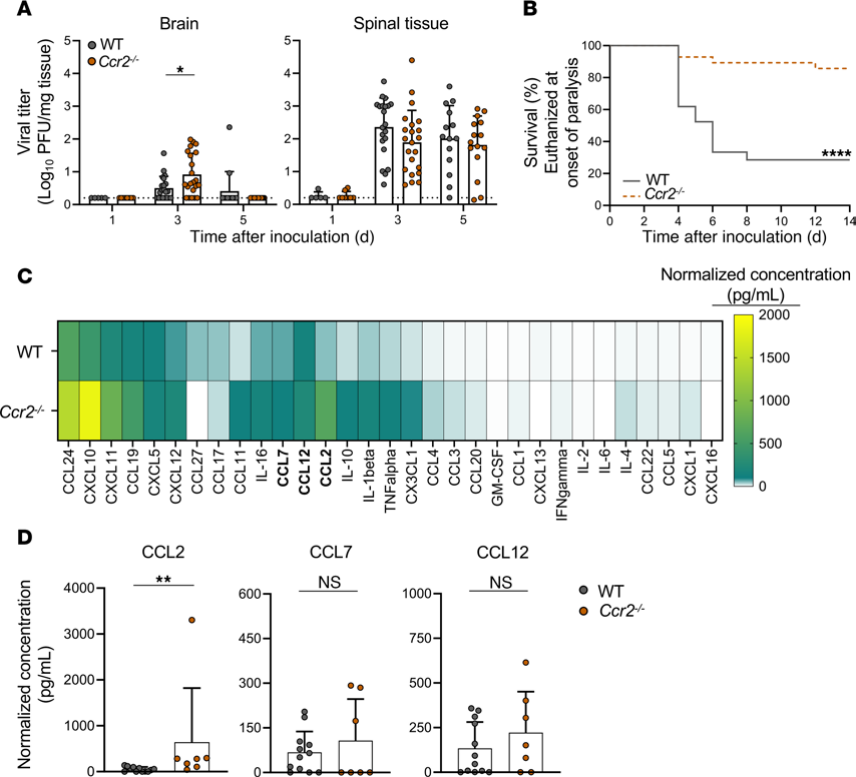
Mice lacking CCR2 develop less paralysis relative to WT mice following inoculation with neurovirulent EV-D68. Three-day-old WT or *Ccr2^–/–^* mice were inoculated i.c. with PBS (mock) or EV-D68 IL52. (**A**) Brain and spinal tissue of virus-inoculated mice were resected at 1, 3, or 5 dpi, and viral titers were determined by plaque assay. Dotted lines indicate the limit of detection. (**B**) Virus-inoculated mice were monitored daily and euthanized upon signs of paralysis. *n* = 21–28 mice per group. (**C**) Spinal tissue was resected 3 dpi and analyzed by Luminex protein assay. Data from WT mice are the same as those from the IL52-inoculated mice represented in Figure 2A, as these experiments were conducted concurrently. Cytokine concentrations are presented as mean values in pg/mL from 3–12 mice and shown as a heatmap normalized to mock-inoculated mice. The CCL2 outlier in the *Ccr2^–/–^* cohort was excluded from statistical analysis but left on graph for transparency. (**D**) Levels of CCL2, CCL7, and CCL12 are shown. Data are representative of 2–3 independent experiments. Each symbol represents an individual mouse. Error bars indicate mean ± SD. Mann-Whitney *U* test (**A** and **D**) or log-rank test (**B**): *P ≤ 0.05; **P ≤ 0.01; ****P ≤ 0.0001.

As EV-D68 IL52–inoculated mice had higher levels of chemoattractant cytokines and increased immune cell infiltrates relative to controls, we hypothesized that cytokine-induced immune cell recruitment influences EV-D68–mediated paralysis. To determine whether lymphocytes recruited to the spinal cord regulate disease, we assessed EV-D68 replication and virulence in mice lacking C-C chemokine receptor type 2 (*Ccr2^–/–^*), which mediates immune cell recruitment. CCR2 is a common receptor used by CCL2, CCL7, and CCL12 (27), which are upregulated by neurovirulent EV-D68 IL52. We inoculated WT or *Ccr2^–/–^* mice with EV-D68 IL52; resected brain and spinal tissues at 1, 3, and 5 dpi; and quantified viral loads by plaque assay. We found no statistically significant differences in viral loads in spinal tissue of WT and *Ccr2^–/–^* mice and only a modest increase in viral loads in brain tissue of *Ccr2^–/–^* mice at 3 dpi (Figure 5A). Despite the similar viral loads, *Ccr2^–/–^* mice were significantly less likely to develop paralysis relative to WT mice (Figure 5B) and did not manifest an observable pattern of paralyzed limb distribution (Supplemental Figure 2B). To define the immune response to EV-D68 IL52 infection in WT and *Ccr2^–/–^* mice, we conducted Luminex-based assays for proinflammatory cytokines in spinal tissue of infected mice (Figure 5C). There were increased levels of CCL2 but not CCL7 or CCL12 in the spinal cords of *Ccr2^–/–^* relative to WT mice (Figure 5D). Collectively, these data suggest that CCR2-deficient mice are markedly protected against EV-D68 disease.

### Immune cell recruitment following EV-D68 infection is impaired in Ccr2^–/–^ mice.

**Figure 6:**
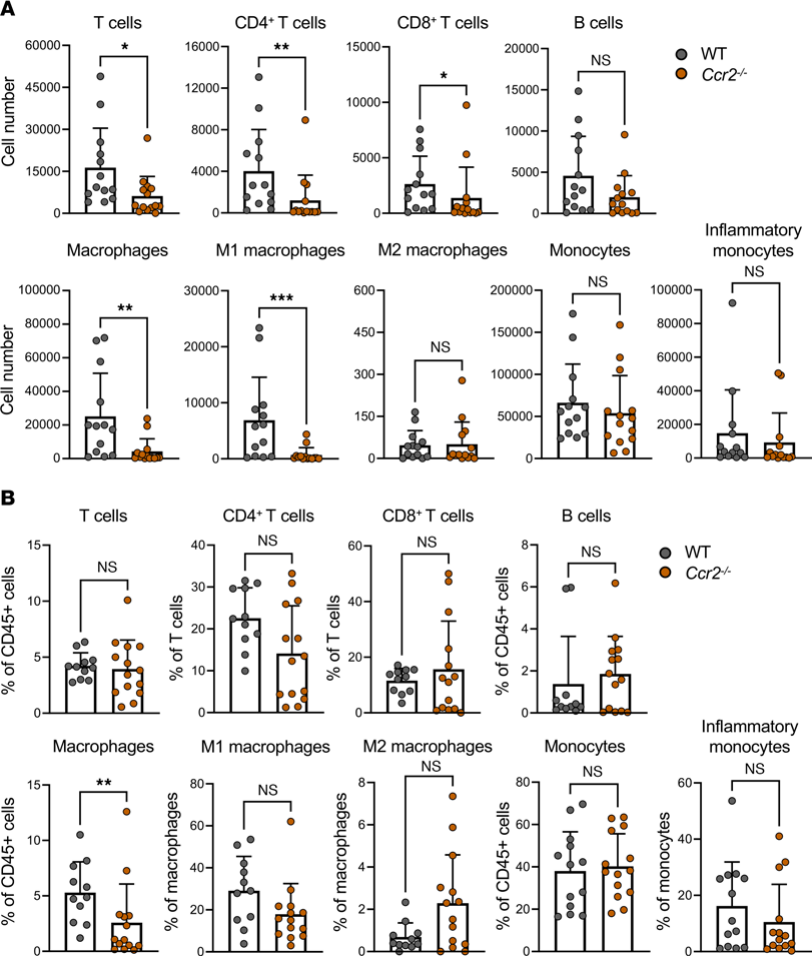
Loss of CCR2 results in altered immune cell recruitment following neurovirulent EV-D68 inoculation. Three-day-old WT or *Ccr2^–/–^* mice were inoculated i.c. with EV-D68 IL52. Spinal cords were resected at 5–6 dpi from paralyzed WT mice or day-matched paralyzed and nonparalyzed *Ccr2^–/–^* mice. Single-cell suspensions were prepared, stained, and analyzed by flow cytometry. (**A**) Numbers and (**B**) percentages of selected cell types are shown. Each symbol represents an individual mouse. Error bars indicate mean ± SD. Mann-Whitney *U* test: *P ≤ 0.05; **P ≤ 0.01; ***P ≤ 0.001.

Since significantly fewer *Ccr2^–/–^* mice developed paralysis following EV-D68 infection than did WT mice, we sought to define differences in the spinal cord immune cell profiles of *Ccr2^–/–^* and WT mice. Spinal cords were resected from EV-D68 IL52–inoculated WT mice that displayed signs of paralysis or day-matched infected *Ccr2^–/–^* mice and analyzed by flow cytometry. Spinal cords of WT mice had increased numbers of total T cells, CD4^+^ T cells, CD8^+^ T cells, total macrophages, and M1 macrophages relative to *Ccr2^–/–^* mice (Figure 6A). Additionally, there was an increased percentage of total macrophages in the spinal cords of WT relative to *Ccr2^–/–^* mice (Figure 6B). Other immune cell populations varied modestly or did not differ significantly between WT and *Ccr2^–/–^*mice (Supplemental Figure 5, A and B). We conclude that CCR2-deficient mice had impaired immune cell recruitment to the spinal cord relative to WT mice following EV-D68 infection.

### Paralysis frequency following EV-D68 infection is diminished in Rag1-deficient mice.

**Figure 7:**
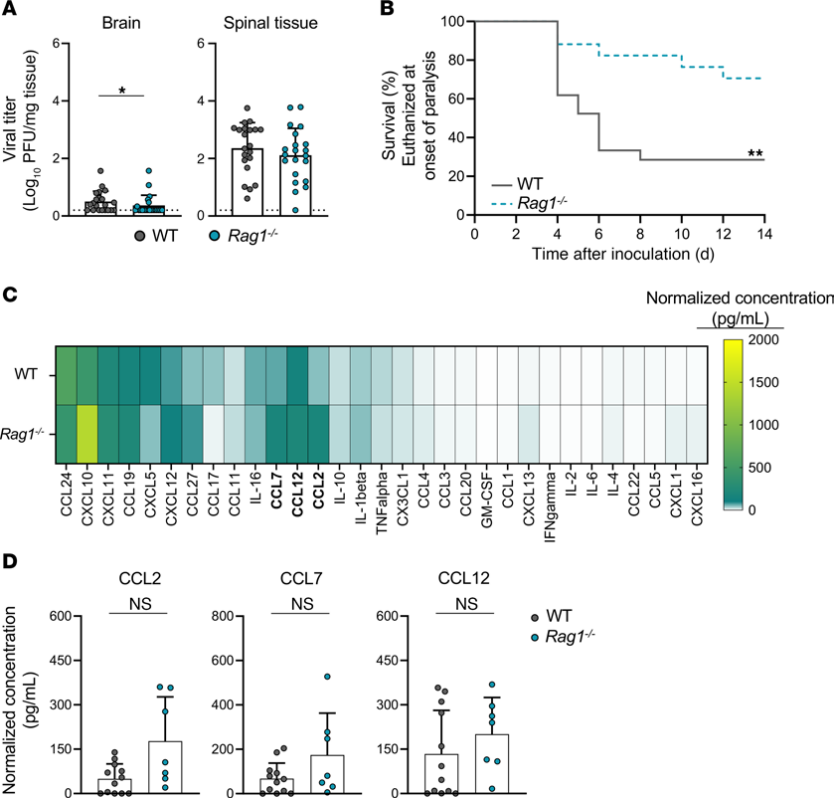
Mice lacking mature B and T cells have diminished EV-D68–induced paralysis relative to WT mice. Three-day-old WT or *Rag1^–/–^* mice were inoculated i.c. with PBS (mock) or EV-D68 IL52. (**A**) Brain and spinal tissue were resected at 3 dpi, and viral titers were determined by plaque assay. Data from WT mice are the same as those presented in Figure 5A, as these experiments were conducted concurrently. Dotted lines indicate the limit of detection. (**B**) Virus-inoculated mice were monitored daily and euthanized upon signs of paralysis. *n* = 16–21 mice per group. Data from WT mice are the same as those presented in Figure 5C, as these experiments were conducted concurrently. (**C**) Spinal tissue was resected 3 dpi and analyzed by Luminex protein assay. Data from WT mice are the same as those presented in Figure 2A, as these experiments were conducted concurrently. Cytokine concentrations are presented as mean values in pg/mL from 7–12 mice and shown as a heatmap normalized to concentrations in mock-inoculated mice. (**D**) Levels of CCL2, CCL7, and CCL12 are shown. Data are representative of 2–3 independent experiments. Each symbol represents an individual mouse. Error bars indicate mean ± SD. Mann-Whitney *U* test (**A** and **D**) or log-rank test (**B**): *P ≤ 0.05; **P ≤ 0.01.

T cells were robustly recruited to the spinal cord of WT mice following infection with EV-D68. Since T cells contribute to disease severity following infection with other neurovirulent viruses (15, 16), we hypothesized that immune cells contribute to acute EV-D68 disease in mice. To determine whether B and T lymphocytes affect EV-D68 replication and virulence, we inoculated either WT mice or *Rag1^–/–^* mice, which lack mature B and T lymphocytes, with EV-D68 IL52 and quantified viral loads at 3 dpi by plaque assay (Figure 7A). There was a modest decrease in viral load in the brains of *Rag1^–/–^* mice relative to WT mice, but there was no significant difference in viral load in spinal tissue. Strikingly, *Rag1^–/–^* mice inoculated with EV-D68 IL52 were significantly less likely to develop paralysis compared with WT mice (Figure 7B), and no infected *Rag1^–/–^* mice had multi-limb paralysis (Supplemental Figure 2B). *Rag1^–/–^* mice had no detectable neutralizing antibodies at 14 dpi, consistent with the absence of mature lymphocytes in these animals (Supplemental Figure 6A). However, *Rag1^–/–^* mice had detectable virus in spinal tissue at 14 dpi, whereas WT and *Ccr2^–/–^* mice had no detectable virus at this time point (Supplemental Figure 6B). Furthermore, IL-52–inoculated *Rag1^–/–^* mice had detectable virus in spinal tissue, liver, and spleen at 14 dpi (Supplemental Figure 6C) and continued to develop paralysis after 14 dpi (Supplemental Figure 6D), whereas WT mice had no detectable virus at 14 dpi, nor did they develop paralysis after this time point. These findings suggest that the failure to clear infection by 14 dpi leads to disease. To determine whether the absence of lymphocytes confers susceptibility to other strains of EV-D68, we inoculated *Rag1^–/–^* mice with MO49 and observed little detectable virus at 3 dpi (Supplemental Figure 6E) and no paralysis (Supplemental Figure 6F). These results indicate that while lymphocytes aid in clearing virus, they also promote EV-D68 paralysis.

To characterize the cytokine response elicited by EV-D68 in WT and *Rag1^–/–^* mice, we conducted Luminex-based assays for proinflammatory cytokines in spinal tissue of mice inoculated with EV-D68 IL52 (Figure 7C). There were no statistically significant differences in the levels of CCL2, CCL7, or CCL12 in the spinal tissue of *Rag1^–/–^* and WT mice following inoculation with EV-D68 IL52 (Figure 7D). These observations suggest that lymphocytes are not required for the cytokine response to EV-D68 in the spinal cord.

To define the types of immune cells recruited into spinal tissue of *Rag1^–/–^* mice inoculated with EV-D68, we used flow cytometry to analyze immune cell subtypes. Spinal tissue of *Rag1^–/–^* mice inoculated with EV-D68 IL52 had increased numbers of total macrophages, M1 macrophages, and inflammatory monocytes relative to spinal tissue of *Rag1^–/–^* mice inoculated with PBS (Supplemental Figure 7A). Additionally, there was an increased percentage of CD4^+^ T cells, total macrophages, M2 macrophages, total monocytes, and inflammatory monocytes in spinal tissue of EV-D68 IL52–inoculated *Rag1^–/–^* mice relative to those inoculated with PBS (Supplemental Figure 7B). Levels of other immune cell types varied modestly or did not differ significantly between EV-D68– and PBS–inoculated *Rag1^–/–^* mice (Supplemental Figure 8). These data suggest that mice lacking mature B and T lymphocytes have an altered immune cell population in spinal tissue following EV-D68 IL52 inoculation.

### Antibody-mediated T cell depletion attenuates EV-D68–mediated paralysis.

**Figure 8:**
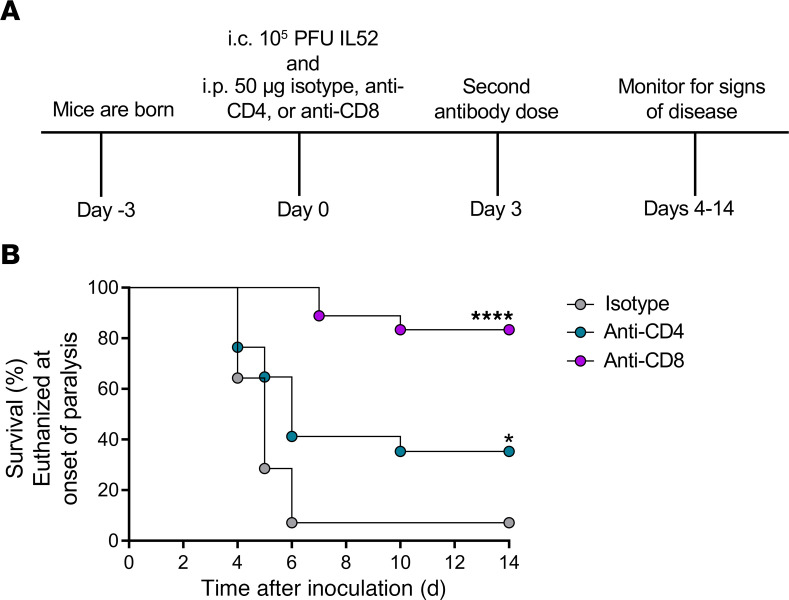
Antibody-mediated depletion of T cells protects mice from EV-D68–mediated paralysis. Three-day-old WT mice were inoculated i.c. with EV-D68 IL52 and subsequently inoculated i.p. with anti-CD4, anti-CD8, or isotype control antibody. Mice received a second dose of antibody at 3 dpi. Mice were monitored daily for signs of disease and euthanized upon detection of paralysis. (**A**) Experimental workflow. (**B**) Percent survival (to day of paralysis onset) following treatment with either isotype control antibody or with anti-CD4 or anti-CD8 antibodies. *n* = 14–18 mice per group. Data are representative of 2 independent experiments. Log-rank test: *P ≤ 0.05; ****P ≤ 0.0001.

Since B cells are not efficiently recruited to the spinal cord following EV-D68 infection, we anticipated that the delay in onset of paralysis of *Rag1^–/–^* mice was attributable to the absence of T cells. To determine whether T cells influence EV-D68 disease, we administered depleting antibodies specific for CD4^+^ or CD8^+^ to WT mice prior to inoculation with EV-D68 IL52 and assessed differences in disease penetrance (Figure 8A). Administration of antibodies against CD8^+^ T cells, and to a lesser extent CD4^+^ T cells, substantially protected mice from paralysis relative to those treated with an isotype antibody control (Figure 8B). The mean day of paralysis onset was approximately 4.8 dpi for control isotype antibody, 5.4 dpi for anti-CD4 antibody, and 8.0 dpi for anti-CD8 antibody. These results indicate that T cells, specifically CD8^+^ T cells, promote EV-D68 paralysis and potentially identify new therapeutic targets against EV-D68–associated AFM.

## Discussion

In this study, we examined the influence of host immunity on CNS replication of EV-D68 and development of paralysis in newborn mice. Infection with a neurotropic EV-D68 strain led to higher levels of spinal tissue cytokines. These cytokines promoted recruitment of inflammatory cell types to the spinal cord, which is considered an immune-privileged tissue (28). Mice lacking functional immune cell recruitment or functional lymphocytes had diminished paralysis relative to immunocompetent mice. These data indicate that immune cells contributed to development of EV-D68–associated paralysis in newborn mice, a finding that enhances understanding of enterovirus neuropathogenesis and provides potential insights into the mechanisms by which host immunity contributes to disease.

Different strains of EV-D68 produced little to no viral load in the brain at 3 dpi in WT mice (Figure 1A), despite the brain being the site of inoculation. The failure of EV-D68 to replicate efficiently in the brain might be attributable to limited expression of proviral host factors, such as receptors or entry mediators, or to a restrictive innate immune response, as replication of EV-D68 is more efficient in the brain of mice lacking receptors for type I interferons (25). EV-D68 IL52 was the only strain tested that produced detectable viral loads in spinal tissue of WT mice at 3 dpi (Figure 1A), and the titers of this strain increased in spinal tissue over time (Figure 1B). At 5 dpi, viral loads in spinal tissue of WT mice varied by over 1,000-fold, raising the possibility that viral titers did not reach the threshold to cause paralysis in 30%–40% of WT mice. Furthermore, differences in host immune determinants also may contribute to differences in disease susceptibility. While *Ccr2^–/–^* and *Rag1^–/–^* mice were less susceptible to EV-D68–mediated paralysis than WT mice, viral loads in spinal tissue of WT and knockout mice at 3 dpi were comparable. These findings suggest that viral replication is necessary but not sufficient for development of paralysis in newborn mice.

The MO49 strain can be virulent under certain conditions. A major mediator of differences in virulence of MO49 and IL52 is an isoleucine-to-valine polymorphism at position 88 in VP3, with minor contributors being a leucine-to-proline polymorphism at position 1 in VP1 and a histidine-to-arginine polymorphism at position 47 in protein 3A (25). We observed that IL52 but not MO49 replicated in WT mice and caused disease following IC inoculation, as observed previously (25). However, intraperitoneal inoculation of AG129 mice, which lack receptors for type I and II interferons, with MO49 leads to acute flaccid paralysis, muscle atrophy, myelitis, and myositis (29). Furthermore, mouse-adapted MO49 replicates in AG129 mice, elicits a robust immune response, and causes paralysis when inoculated intranasally (29, 30). Although the MO49 strain used in our study was not neurovirulent, it is possible that differences in mouse strain, inoculation route, or viral sequence influenced the outcome of these experiments. However, in both the previous and current studies, EV-D68 infection led to induction of MCP-1 (CCL2 in the mouse cytokine panel), suggesting that this cytokine is a common response to EV-D68 across tissue types. Both MO49 and IL52 replicate in mouse cortical neurons and organotypic brain slice cultures (21). A caveat of these studies is the use EV-D68 clinical-isolate strains with an unclear passage history, which raises the possibility that these strains vary from EV-D68 strains derived from infectious cDNA clones. Nonetheless, these studies suggest that MO49 is neurovirulent in certain circumstances and may provide a framework for future studies of viral and host determinants of EV-D68 pathogenesis.

EV-D68 is rarely detected in the CSF of children with AFM, with only 1 of 55 CSF specimens collected in 2014 having evidence of the virus (6). In mice, disease is attributed to loss of motor neurons in the spinal cord, as well as myositis (29). In an epidemiological study of children with AFM, 13% had myositis, providing evidence that myositis is associated with infection by some non-polio enteroviruses (31). While our study focused on the influence of host immunity in the spinal cord of mice, future studies examining EV-D68 replication and inflammatory responses in muscle may shed additional light on EV-D68 disease.

EV-D68 infection of mice and hSCOs elicited a cytokine response. In both mice and organoids, IL52 led to generally higher levels of cytokines than did MO49 (Figure 2B and Figure 3B), although the differences in cytokine levels induced by these strains in mice are more modest. MO49 was associated with higher levels of CCL11 in mice (Figure 2B) and IL-4 and IL-10 in organoids (Figure 3B), suggesting virus strain–specific differences in cytokine induction. Given that MO49 and IL52 differed in the frequency of paralysis following infection of mice (Figure 1D), we focused on differences in the cytokines induced by these strains and observed that levels of CCL2 (MCP-1 in the human organoid cytokine panel), CCL7, and CCL12 were substantially higher following infection with paralysis-inducing strain IL52. Mice lacking CCR2, the common receptor used by these cytokines, developed paralysis significantly less frequently than did WT mice following IL52 inoculation (Figure 5B). These data suggest that an IL52-induced cytokine response contributes to immune cell recruitment and neuropathogenesis.

Inflammation contributes to host immunity and can limit viral replication and disease, but it may augment disease if unregulated or it occurs in an immune-privileged site (32). Compared with mice inoculated with MO49 or PBS, mice inoculated with IL52 elaborated higher levels of chemoattractant cytokines (Figure 2), which mediate immune cell recruitment by binding to CCR2 (33). CCR2 is expressed by several cell types, including dendritic cells, macrophages, monocytes, B cells, T cells, and other immune and nonimmune cells (27, 33). Significantly greater numbers of CD8^+^ T cells and macrophages were present in the spinal cord of mice inoculated with IL52 relative to those inoculated with MO49 or PBS (Figure 4). This robust immune cell infiltration elicited by IL52 is similar to that observed in postmortem studies of a child who had flaccid paralysis, in which macrophages, CD8^+^ T cells, and perforin but not caspase-3 were detected in spinal cord sections (3, 8). These observations suggest that the immune response, and not virus-induced apoptosis, contributes to EV-D68 neuropathogenesis.

Mature lymphocytes can impair recovery following spinal cord injury and promote damage to myelin (17). Since antibody-mediated depletion of CD8^+^ T cells significantly reduced paralysis in IL52-infected mice, we conclude that immune-mediated cytotoxicity contributes to motor neuron injury and development of paralysis. T cell recruitment to the spinal cord was influenced by CCR2 expression, as lack of CCR2 resulted in diminished numbers of T cells in the spinal cord during EV-D68 infection (Figure 6). However, T cells express modest levels of CCR2 relative to other immune cells, suggesting that T cell recruitment could be influenced directly by CCR2 or indirectly by other CCR2-expressing immune cells such as inflammatory monocytes (34). MHC class I molecules are expressed by neurons during mid-gestation (E9.5–E10.5) (35–37). Thus, EV-D68–infected neurons presenting viral peptides in complex with MHC class I could be recognized by CD8^+^ T cells entering spinal tissue and killed by production of cytolytic molecules such as perforin and granzyme B (38).

Three-day-old WT mice inoculated with strain IL52 developed paralysis by 5–6 dpi and had significantly greater numbers of T cells in spinal tissue than those inoculated with strain MO49 or PBS (Figure 4), suggesting a rapid T cell response to EV-D68 despite the young age of the mice. Functions of T cells in newborn and adult mice differ (39–41), which may explain this early T cell response to EV-D68 in our experiments. In humans, neonatal CD8^+^ T cells have a distinct pattern of gene expression relative to adult CD8^+^ T cells (42). In newborn mice, naive CD8^+^ T cells are more functionally reactive, proliferate rapidly, and become terminally differentiated (43). Since newborn WT mice develop paralysis as early as 4 dpi, T cells may function in a manner independent of antigen. In addition to MHC class I–restricted recognition of EV-D68 peptides, newborn CD8^+^ T cells might manifest nonspecific innate-like immune functions that lead to neuronal cell death (39, 42). Together, these findings raise the possibility that T cells also contribute to AFM in humans. However, the mechanisms by which T cells contribute to AFM in humans and mice may differ.

Antibody-mediated depletion of T cells provided substantial but not complete protection against EV-D68–mediated disease, suggesting that additional immune cell subsets contribute to development of paralysis. We think it possible that a multicellular inflammatory environment leads to EV-D68–associated AFM. Other inflammatory immune cells, such as CD86^hi^/iNOS^hi^ M1 macrophages and Ly6C^hi^ monocytes, which express CCR2 and were detected in spinal tissue of paralyzed mice, could influence disease by directly or indirectly injuring neurons (17, 44, 45). Understanding the contribution of immune cells and their mechanisms of neuronal injury may aid in identifying more specific immune-related therapies during the acute presentation of AFM.

Current treatments for EV-D68–mediated AFM are limited. There is insufficient evidence to support the use or avoidance of any specific therapy (46). Treatment of EV-D68–infected mice with dexamethasone, a corticosteroid, is associated with significantly higher loads of EV-D68 and enhanced disease (9). Fluoxetine, an FDA-approved antidepressant, reduces EV-D68 replication in cell culture (47) but has no effect on viral loads in the spinal cord or EV-D68–induced disease in mice and is not associated with improved outcomes in a multicenter cohort study of individuals with AFM (9, 47). Human intravenous immunoglobulin containing EV-D68–neutralizing antibodies reduces viral loads and disease in mice, but only if administered early in the disease course (9). Other antiviral medications, including pleconaril, pocapavir, and vapendavir, lack significant activity against contemporary strains of EV-D68 at clinically relevant concentrations (48). Although vaccination has been a successful strategy for prevention of poliomyelitis, the large number of potentially paralytogenic enteroviruses make development of vaccines to prevent AFM a challenging undertaking (49). Since administration of T cell–specific antibodies significantly protected mice against EV-D68–associated paralysis, therapeutics targeting T cell recruitment, CNS entry, or function may be an efficient strategy to treat EV-D68 neurologic disease in humans.

This study has several limitations. Intracranial inoculation, while serving as a more neuropathogenic entry route in mice, is not the natural route of infection in any species, including humans. We conducted the majority of the experiments using a single strain of EV-D68 and highly-susceptible newborn mice. Examining the influence of immune cells in EV-D68 disease using different routes of inoculation, a variety of EV-D68 strains, and mice beyond the neonatal interval will provide a broader understanding of how host immunity influences EV-D68 disease.

Our experiments show that inoculation of newborn mice with a neurotropic strain of EV-D68 resulted in higher levels of chemoattractant cytokines in spinal tissues. These cytokines promoted immune cell recruitment to the spinal cord and, in turn, the immune cells promoted paralysis. Administration of antibodies specific for either CD4^+^ or CD8^+^ T cells diminished development of EV-D68–mediated paralysis in newborn mice, with a greater effect observed with CD8^+^ depletion. There are currently no FDA-approved virus-specific therapeutics or vaccines to prevent EV-D68–mediated AFM (43). Our findings provide insights about the influence of host immunity on EV-D68 replication and pathogenesis in the CNS and illuminate potential targets for therapeutic intervention.

## Methods

### Sex as a biological variable.

Multiple litters were used for each experiment to diminish any possible sex-based influence, although sex was not anticipated to contribute to disease outcomes due to the very young age of the mice.

### Cells and viruses.

Rhabdomyosarcoma (RD) cells (ATCC, CCL-136) and human lung bronchial epithelial BEAS-2B cells (ATCC, CRL-3588) were propagated at 37°C and 5% CO_2_ in DMEM supplemented to contain 10% FBS, 100 U/mL penicillin, and 100 μg/mL streptomycin. Infectious cDNAs of US/MO/14-18949 (MO49) and US/IL/14-18952 (IL52) were provided by Raul Andino (UCSF, San Francisco, California, USA). The following reagents were obtained through BEI Resources, National Institute of Allergy and Infectious Diseases (NIAID), NIH: plasmid pUC19 containing cDNA from EV-D68, USA/Fermon infectious clone EV-D68-R-Fermon (NR 52375). Studies with EV-D68 were conducted at biosafety level 2 (BSL-2).

Virus stocks were prepared using RD cells that were previously propagated at 37°C and then shifted to 33°C after cotransfection of the T7 promoter–containing infectious cDNA plasmids and plasmid expressing T7 RNA polymerase (50). T7opt in pCAGGS was a gift from Benhur Lee (Addgene plasmid 65974). When cytopathic effect (CPE) was evident (3–5 dpi), cells were frozen and thawed 3 times, and lysates were adsorbed to RD cells for 24 hours or until CPE was observed. Cells were collected by scraping, resuspended in a small volume of PBS, frozen and thawed 3 times, and clarified by centrifugation to prepare high-titer viral stocks.

### Viral plaque assays.

Viral titers in cell-culture lysate stocks and tissue homogenates were determined by plaque assay using RD cells that were previously propagated at 37°C and then maintained at 33°C for the duration of the viral plaque assay. Serial 10-fold dilutions of samples in sterile PBS containing calcium and magnesium were adsorbed to RD cells at room temperature (RT) for 1 hour. Monolayers were overlaid with a 1:1 (vol/vol) mixture of 2% agarose (Invitrogen, 16500500) and 2X MEM (Gibco) supplemented to contain 20% FBS, 200 U/mL penicillin, and 200 μg/mL streptomycin and incubated at 33°C for 48 hours. Plaques were visualized and enumerated following staining with neutral red.

### Mouse experiments.

Mouse experiments were conducted in animal BSL-2 facilities. Three-day-old mice were inoculated intracranially (i.c.) in the right cerebral hemisphere with 5 µL containing 10^5^ PFU of EV-D68 diluted in sterile PBS using a 30-gauge needle and syringe (Hamilton). For survival experiments, mice were monitored daily for signs of disease. Death was not used as an endpoint; mice that had signs of paralysis (e.g., limb dragging and unresponsiveness to stimuli) were euthanized immediately with isoflurane. We inoculated 3-day-old mice at approximate sex ratios of 1:1. We used multiple litters for each experiment to diminish any possible sex-based differences in experimental outcomes.

For viral replication experiments, mice were euthanized at various intervals after inoculation, and tissues were collected, weighed, and suspended in 1 mL PBS. Tissues were homogenized by mechanical disruption with stainless steel beads using a TissueLyser (QIAGEN) and frozen and thawed 3 times. Viral titers in tissue homogenates were determined by plaque assay using RD cells. Titers were normalized to the weight of each tissue. For viral growth experiments, BEAS-2B cells (2 × 10^5^/well) were seeded into 24-well plates, incubated overnight, and adsorbed with EV-D68 in PBS at an MOI of 2 PFU/cell at RT for 1 hour. The inoculum was removed, and cells were incubated in 0.5 mL fresh medium at 33°C. At 0, 8, or 24 hours after adsorption, plates were frozen and thawed twice prior to determination of viral titer by plaque assay using RD cells.

For antibody depletion experiments, 3-day-old mice were inoculated i.c. as described and administered 50 μg anti–keyhole limpet hemocyanin IgG2b isotype (Bio X Cell, BE0090), anti-CD4 clone GK1.5 (Bio X Cell, BE0003-1), or anti-CD8 clone 2.43 (Bio X Cell, BE0061) intraperitoneally in a volume of 30 mL sterile *InVivo*Pure dilution buffers recommended for each clone (Bio X Cell). Mice were administered a second antibody dose 3 days after the first and were monitored for signs of disease.

### Serum neutralization.

Blood was obtained from euthanized mice and allowed to coagulate at RT for 30 minutes. Sera were obtained by clarifying coagulated blood and heat inactivated at 56°C for 30 minutes. Sera were serially diluted 1:2 (vol/vol) in complete DMEM, and virus was diluted to a final concentration of 100 TCID_50_ (50% tissue culture infectious dose) per well. Virus was incubated with serum dilutions at 33°C for 1 hour and inoculated onto RD cells. At 5 dpi, cells were fixed and stained with crystal violet.

### hSCO cultivation and infection.

Human iPSCs (SCTi003-A, STEMCELL Technologies, 200-0511) were maintained in mTeSR Plus pluripotent stem cell medium (STEMCELL Technologies, 100-0276) supplemented to contain 10 μM Y-27632 (Tocris, 1254) in flasks coated with 150 μg/mL Cultrex (R&D Systems, 3434-005-02). To prepare 3-DiSC hSCOs, a single-cell suspension of iPSCs was prepared using ACCUMAX (STEMCELL Technologies), and cells were seeded at a density of 9,000 cells/well in 96-well, round-bottom, low-adhesion plates in 100 μL N2B27 differentiation medium (1:1 [vol/vol] DMEM/F-12 [Gibco] and neurobasal medium [Gibco] supplemented to contain 10% KnockOut serum replacement [Invitrogen], 0.5% N2 supplement [Thermo Fisher Scientific], and 1% B27 supplement without vitamin A [Invitrogen] supplemented to contain 1 mM l-glutamine [Gibco], 0.1 mM 2-mercaptoethanol [MilliporeSigma], and 0.5 μM ascorbic acid [MilliporeSigma]) as described previously (11). Every 3 days during differentiation, 50% of the medium was replaced with fresh medium. For patterning, on days 0–3, the medium was supplemented to contain 10 μM Y-27632 (Tocris, 1254), 20 ng/mL bFGF (Thermo Fisher Scientific, 13256-029), and 3 μM CHIR 99021 (Tocris, 4423). From day 0 to 6, the medium was supplemented to contain 10 μM SB431542 (Tocris, 1614). From day 3 to 15, the medium was supplemented to contain 100 nM retinoic acid (Tocris, 302-79-4) and 500 nM smoothened agonist (SAG; Tocris, 912545-86-9).

For infection experiments, hSCOs were plated in pools of 8–12 organoids per well and inoculated with PBS or virus (10^5^ PFU/pool) at RT for 1 hour. Organoids were washed 3 times with PBS and transferred to a new well prior to incubation with fresh medium at 33°C for the duration of the experiment.

### Immunofluorescence.

Antibody staining of hSCOs was conducted using the fructose-glycerol clearing method as described previously (51). hSCOs were fixed with 4% PFA and washed with PBS and 0.1% Tween-20 (vol/vol) supplemented to contain 0.2% (mass/volume) BSA at 4°C. hSCOs subsequently were washed 3 times with organoid wash buffer (OWB) (0.1% Triton X-100 [vol/vol] and 0.2% BSA [mass/volume] in PBS). hSCOs were incubated with VP1-specific antibody (Genetex, GTX132313) and NeuN-specific antibody (BioLegend, 834501) at a 1:250 dilution overnight at 4°C, washed 3 times with OWB, and incubated with goat anti-rabbit IgG (Thermo Fisher Scientific, A-11008) and goat anti-mouse IgG (Thermo Fisher Scientific, A-11001) at a 1:1,000 dilution overnight at 4°C. hSCOs were cleared with 60% glycerol in 2.5 M fructose for 30 minutes, mounted on slides, and imaged using a Leica Stellaris 5 confocal microscope. Images were processed using Fiji (52).

Mice were inoculated with either PBS or EV-D68 IL52 and euthanized when paralysis was first detected in the IL52-inoculated animals. Spinal columns were dissected and fixed with 10% formalin at RT for 3 days. Spinal cords were dissected and placed in SpineRacks (53), covered with OCT compound, and frozen. Frozen sections were processed and immunostained as described previously (54). Sections were fixed with 4% formaldehyde at RT for 15 minutes, rinsed 3 times with PBS, permeabilized with 0.1% Triton X-100 in PBS for 10 minutes, rinsed 3 times with PBS, and blocked overnight at 4°C in PBS supplemented to contain 5% BSA. Serial sections were stained with VP1-specific antibody (Genetex, GTX132313) at a 1:250 dilution or NeuN (BioLegend, 834501) at RT for 1 hour; rinsed 3 times with PBS; and stained with goat anti-rabbit IgG (Thermo Fisher Scientific, A-11035) and goat anti-mouse IgG (Thermo Fisher Scientific, A-11001), both at a 1:1,000 dilution at RT for 1 hour. Sections were counterstained with DAPI.

### Luminex assays.

Brain or spinal tissue homogenates prepared from samples used for viral titer determination were analyzed by Luminex profiling with the Bio-Plex Pro Mouse Chemokine 31-plex panel (Bio-Rad, 12009159) according to the manufacturer’s instructions. Cytokine levels were quantified using the LabMAP multianalyte profiling system (Luminex).

Supernatants were collected from hSCOs inoculated with PBS or EV-D68 at 3 dpi, and cytokine levels were determined with the Bio-Plex Human Inflammation Panel 1 37-plex assay kit (Bio-Rad, 171AL001M) according to the manufacturer’s instructions using the MAGPIX laboratory multianalyte profiling system (MilliporeSigma) developed by Luminex.

### Flow cytometric analyses of neonatal mouse spinal cord samples.

Mice were inoculated with either PBS or EV-D68 IL52 and euthanized when paralysis was first detected in the IL52-inoculated animals. Spinal cords were resected and submerged in ice-cold HBSS (Gibco, 14175095). Spinal cords were incubated with 0.5 mg/mL trypsin (Worthington, LS003708) in HBSS at RT for 30 minutes, washed twice with HBSS, and manually dissociated with a fire-polished borosilicate glass Pasteur pipette. Cell suspensions were passed through a 70 μm cell strainer (Falcon, 352350).

Cells were collected to form a pellet by centrifugation at 450***g*** for 4 minutes, resuspended in FACS buffer, and incubated with the antibodies and stains shown in Supplemental Table 1. Single-cell suspensions were washed with PBS and stained with LIVE/DEAD Fixable Violet dye (Thermo Fisher Scientific, L34963) at 1:1,000 in PBS for 15 minutes. Cells were washed with FACS buffer (2% heat-inactivated FBS in PBS) and blocked with CD16/CD32-specific antibody (Tonbo Biosciences, 70-0161-M001) and True-Stain Monocyte Blocker (BioLegend, 46102) according to the manufacturer’s protocol. Cells were stained with antibodies specific for surface proteins (Supplemental Table 1) (1:100, vol/vol) in Brilliant Stain buffer (BD Biosciences, 566349) at 4°C for 30 minutes and washed with FACS buffer. Cells were fixed and permeabilized using Cytofix/Cytoperm solution (BD Biosciences, 554714) at RT for 30 minutes. Cells were stained with antibodies specific for intracellular proteins (Supplemental Table 1) (1:100,vol/vol) in Perm/Wash buffer (BD Biosciences) supplemented to contain 2% rat serum at RT for 1 hour. Cells were washed with Perm/Wash buffer, resuspended in FACS buffer, and analyzed using an Aurora multispectral flow cytometer (Cytek). Absolute cell number was determined by comparison with Precision Count Beads (BioLegend, 424902). Cell populations were identified using a previously described sequential gating strategy (55, 56).

### Statistics.

Differences between groups were assessed using unpaired 2-tailed Student’s *t* tests or log-rank tests. Error bars in figures represent SD of the mean. A *P* value less than 0.05 was considered statistically significant. All analyses of data were conducted using GraphPad Prism (version 10.1.2).

### Study approval.

All animal husbandry and experimental procedures were conducted in accordance with the *Guide for the Care and Use of Laboratory Animals* (National Academies Press, 2011) and approved by the University of Pittsburgh Institutional Animal Care and Use Committee.

### Data availability.

All data used to generate Luminex graphs are provided in the Supporting Data Values file. Request for reagents and protocols should be directed to the corresponding authors.

## Author contributions

MAWA, JL, JVW, MCF, and TSD designed research studies. MAWA, JL, SM, IEB, JEJ, and MCF conducted experiments. MAWA, JL, SM, IEB, JEJ, and MCF acquired data. MAWA, JL, SM, JEJ, JVW, MCF, and TSD analyzed data. MAWA, MCF, and TSD wrote the manuscript.

## Supplementary Material

Supplemental data

Supporting data values

## Version 1 06/03/2025

In-Press Preview

## Version 2 08/01/2025

Electronic publication
