# Supplementary material for: Immune cells promote paralytic disease in mice infected with enterovirus D68
Source: J Clin Invest. 2025 Jun 3;135(15):e188495. doi: 10.1172/JCI188495 (PMC12321388; doi:10.1172/JCI188495)
Supplement: Supplemental data [file jci-135-188495-s052.pdf]

Supplemental Information

Immune cells promote paralytic disease in mice infected with enterovirus D68

Mikal A. Woods Acevedo, Jie Lan, Sarah Maya, Jennifer E. Jones, Isabella E. Bosco, John V. Williams, Megan Culler Freeman, and Terence S. Dermody

Supplemental Figures and Legends

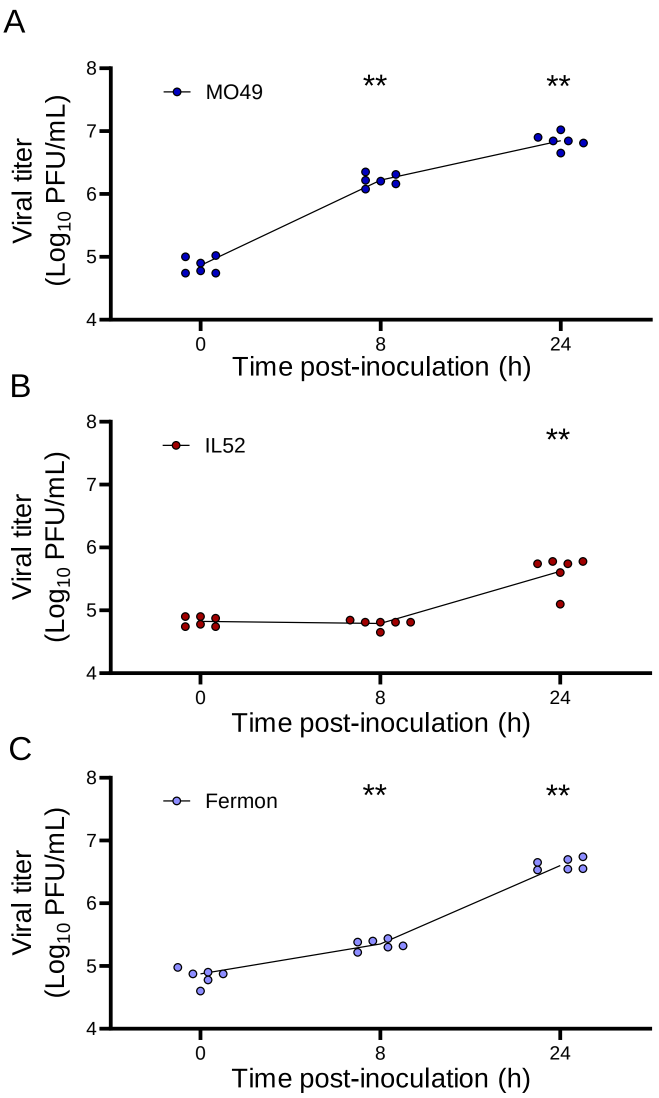

Supplemental Figure 1. EV-D68 strains replicate efficiently in a human respiratory epithelial cell line. BEAS-2B cells were adsorbed with either (A) MO49, (B) IL52, or (C) Fermon at an MOI of 2 PFU/cell. Viral titers in cell lysates at the times post-adsorption shown were determined by plaque assay. Data are representative of 2 independent experiments. Mann-Whitney test: **, $P \leq 0.01$.

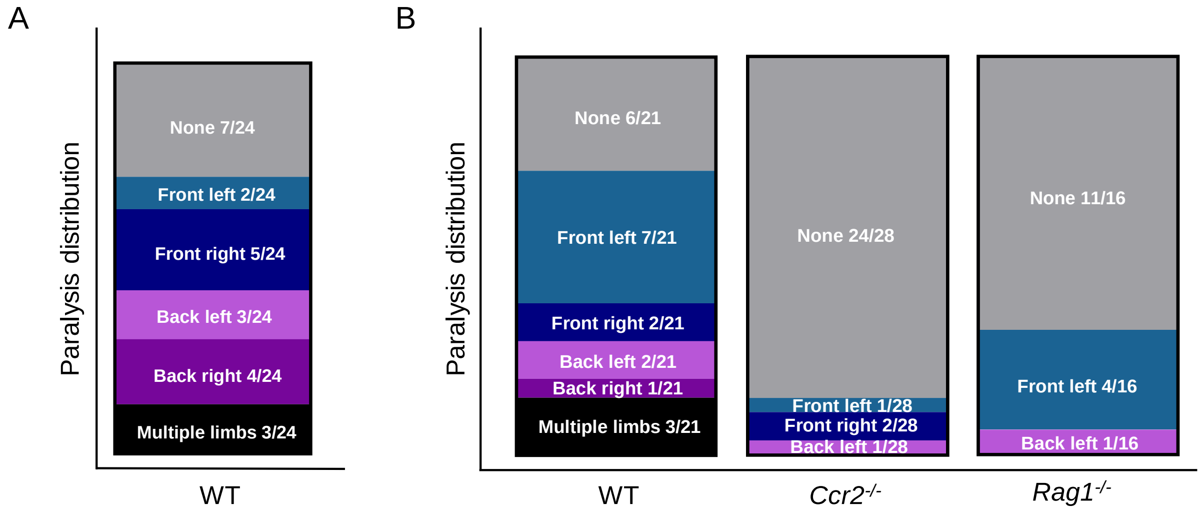

Supplemental Figure 2. Paralyzed limb distribution of US/IL/14-18952-inoculated mice. Three-day-old mice of the indicated genotypes were inoculated i.c. with 10^5 PFU of EV-D68 IL52, monitored daily for disease, and euthanized upon signs of paralysis. Distribution of limb paralysis from experiments presented in (**A**) Figure 1D and (**B**) Figures 5B and 7B. Experiments shown in Figures 5B and 7B were conducted concurrently. Data are representative of 2-3 independent experiments.

Antibody or dye	Color	Clone	Supplier
Viability dye	Live Dead Violet	-	Invitrogen
CD45	BV510	30-F11	BioLegend
CD11b	PE-CF594	M1/70	BD
CD11c	BUV805	HL3	BD
Ly6G	APC-Cy7	1A8	BioLegend
Ly6C	FITC	AL-21	BD
I-A/I-E	AF700	M5/114.15.2	BioLegend
CD24	BUV661	M1/69	BD
CD103	BV785	2E7	BioLegend
XCR1	BV421	ZET	BioLegend
CD3	BV750	17A2	BioLegend
CD4	BUV395	RM4-4	BD
CD19	BV570	6D5	BioLegend
CD8a	AF532	53-6.7	eBioscience
CD172a	BUV737	P84	BD
B220	APC/Fire810	RA3-6B2	BioLegend
CD64	BV711	X54-5/7.1	BioLegend
CD206	BV650	C068C2	BioLegend
CD86	BUV563	GL1	BD
CD93	PerCP/Cy5.5	AA4.1	BioLegend
NK1.1	PE/Cy5	PK136	BioLegend
FceR1α	Super Bright 600	MAR-1	eBioscience
Siglec-F	AF647	S17007L	BioLegend
CD163	PE	TNKUPJ	eBioscience
iNOS	PE/Cy7	CXNFT	eBioscience

Supplemental Table 1. Antibody and dye reagents used for flow cytometry analysis.

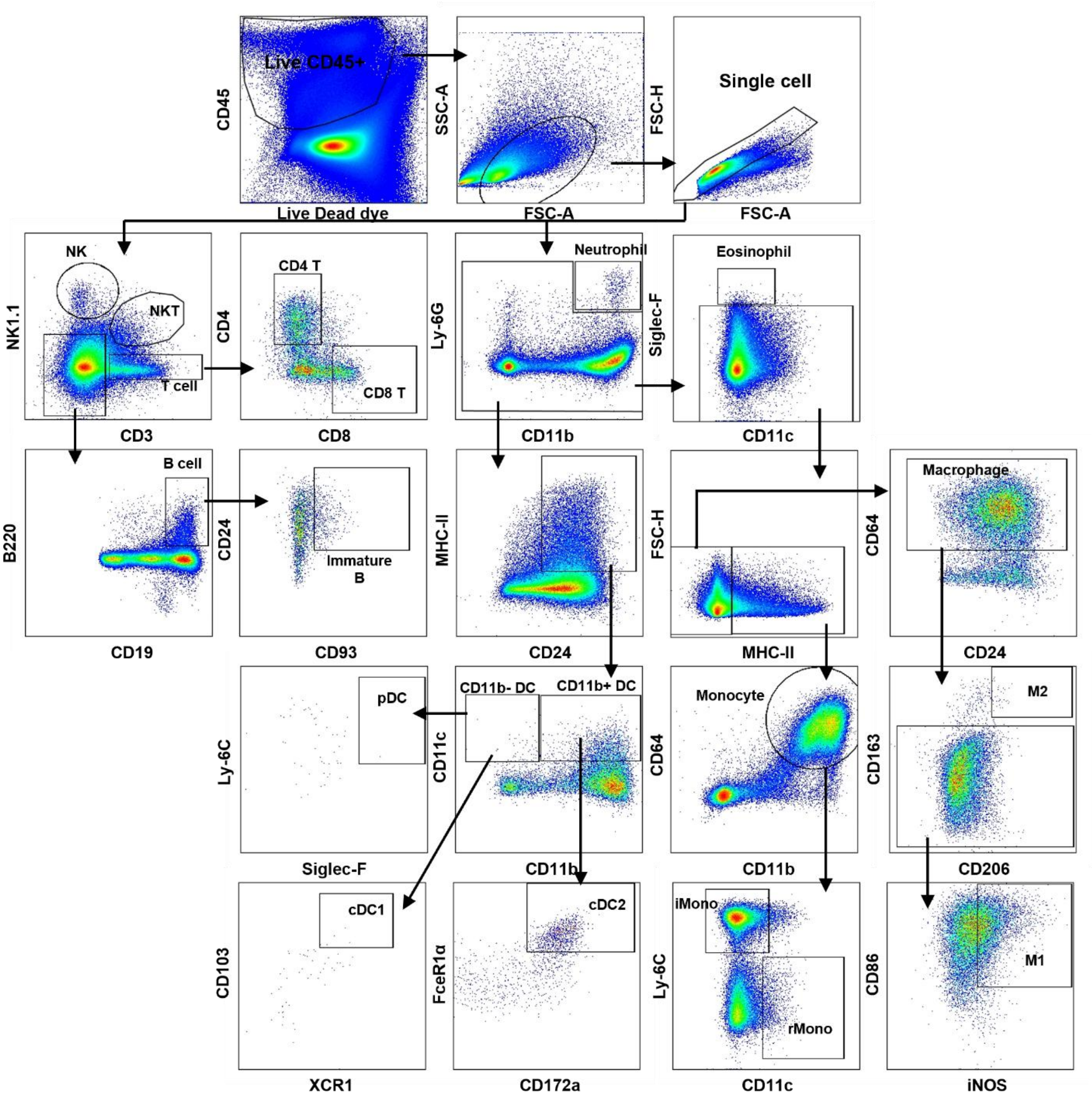

Supplemental Figure 3. Gating strategy used to identify immune cell populations in the spinal cord. Flow cytometric gating strategy for mouse leukocytes in the spinal cord. Frequency and absolute cell numbers of different subpopulations of immune cells were assessed. Representative pseudocolored dot density plots from EV-D68 IL52-inoculated WT mice. Boxed or circled populations labeled with cell-type-specific antibodies indicate populations of interest.

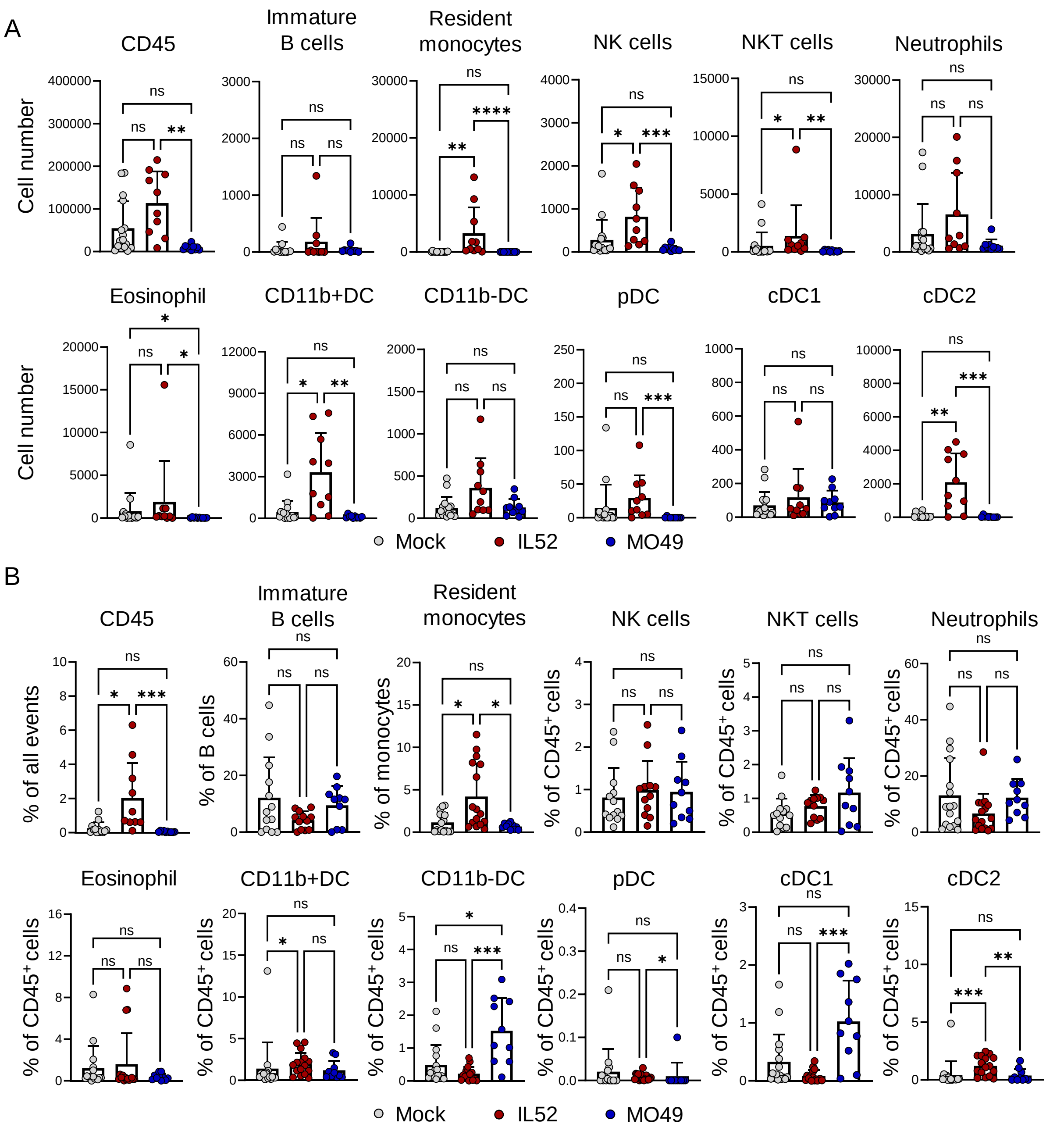

Supplemental Figure 4. Mice inoculated with neurovirulent EV-D68 have altered populations of immune cells in the spinal cord. Three-day-old WT mice were inoculated i.c. with PBS (mock) or EV-D68 MO49 or IL52. Spinal cords were resected from paralyzed IL52-inoculated mice or day-matched mice inoculated with MO49 or PBS. Single-cell suspensions were prepared, stained, and analyzed by flow cytometry. (**A**) Numbers and (**B**) percentages of the indicated cell types are shown. Data are

representative of 2-4 independent experiments. Each symbol represents an individual mouse. Error bars indicate mean ± SD. Kruskal-Wallis test: *, $P \leq 0.05$; **, $P \leq 0.01$; ***, $P \leq 0.001$; ****, $P \leq 0.0001$; ns = not significant.

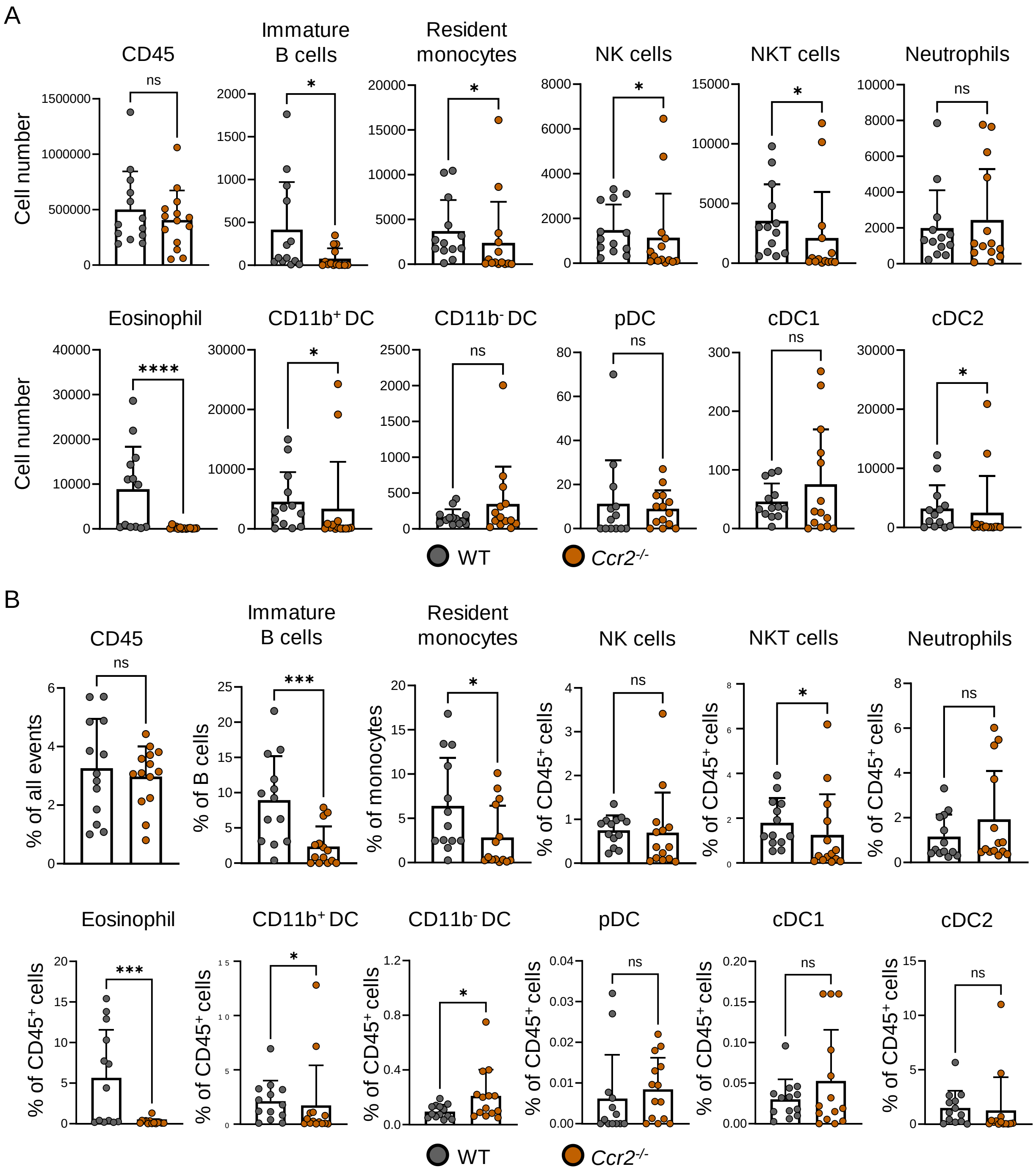

Supplemental Figure 5. *Ccr2*$^{-/-}$ mice have altered immune cell recruitment following neurovirulent EV-D68 inoculation. Three-day-old WT or *Ccr2*$^{-/-}$ mice were inoculated i.c. with EV-D68 IL52. Spinal cords were resected from paralyzed WT mice or day-matched *Ccr2*$^{-/-}$ mice. Single-cell suspensions were prepared, stained, and

analyzed by flow cytometry. (**A**) Numbers and (**B**) percentages of the indicated cell types are shown. Each symbol represents an individual mouse. Error bars indicate mean ± SD. Mann-Whitney test: *, $P \leq 0.05$; **, $P \leq 0.01$; ***, $P \leq 0.001$; ****, $P \leq 0.0001$; ns = not significant.

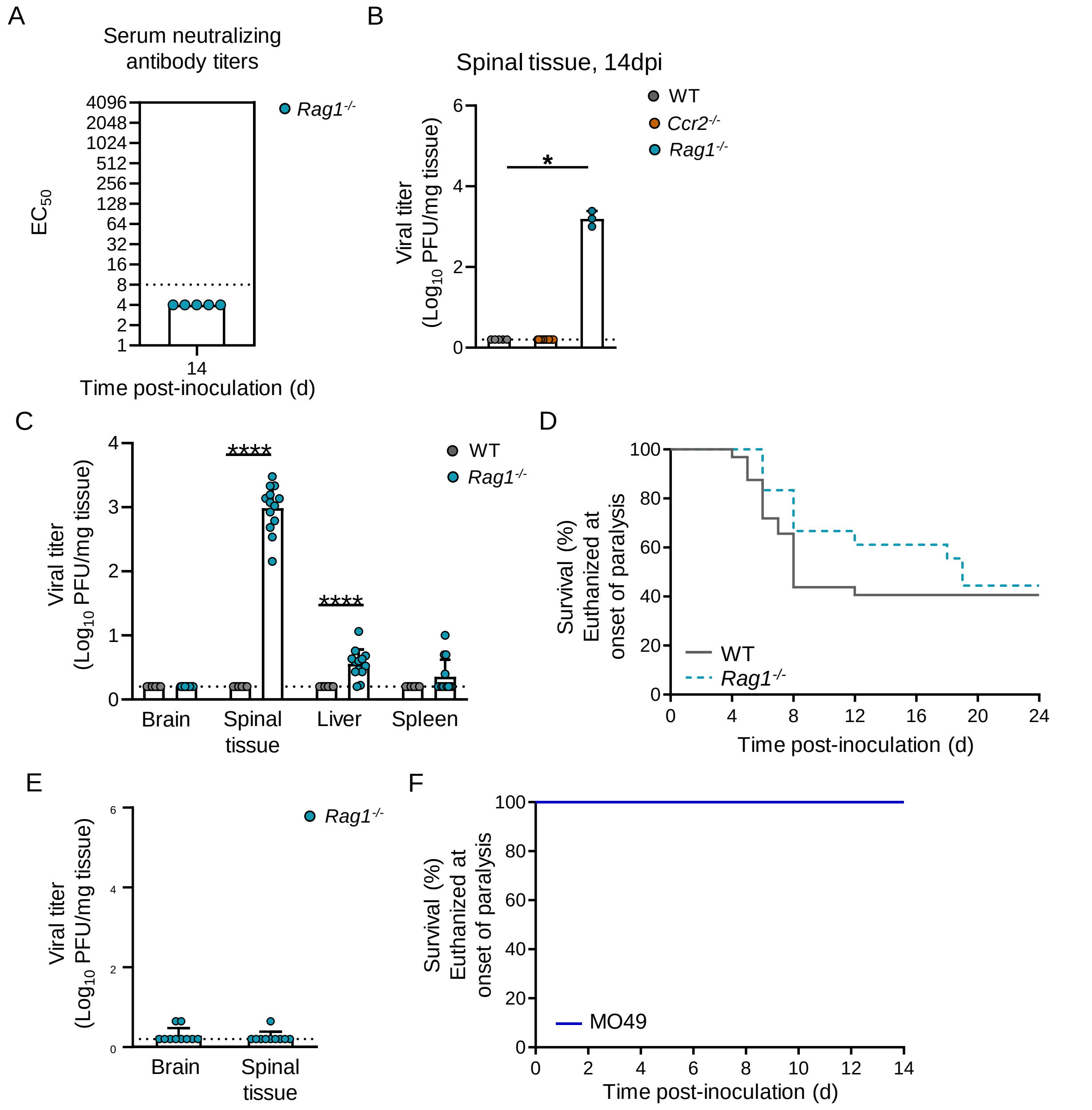

Supplemental Figure 6. EV-D68-inoculated *Rag1*$^{-/-}$ mice fail to produce neutralizing antibodies or clear virus. Three-day-old WT, *Ccr2*$^{-/-}$, or *Rag1*$^{-/-}$ mice were inoculated i.c. with EV-D68 IL52 or MO49. (**A**) Serum was collected at 14 dpi from IL52-inoculated *Rag1*$^{-/-}$ mice and assessed for neutralizing antibody titers. Dotted line indicates the limit of detection. (**B**) Spinal tissue was resected at 14 dpi from IL52-inoculated mice, and viral titers were determined by plaque assay. (**C**) Brain, spinal

tissue, liver, and spleen were resected at 14 dpi from IL52-inoculated WT or *Rag1*$^{-/-}$ mice, and viral titers were determined by plaque assay. (**D**) IL52-inoculated mice were monitored daily for 24 days and euthanized upon signs of paralysis. N = 18-32 mice per group. (**E**) Brain and spinal tissue were resected at 3 dpi from MO49-inoculated *Rag1*$^{-/-}$ mice, and viral titers were determined by plaque assay. (**F**) MO49-inoculated *Rag1*$^{-/-}$ mice were monitored daily for 14 days and euthanized upon signs of paralysis. N = 20 mice per group. Each symbol represents an individual mouse. Mann-Whitney test: *, $P \leq 0.05$; ****, $P \leq 0.0001$.

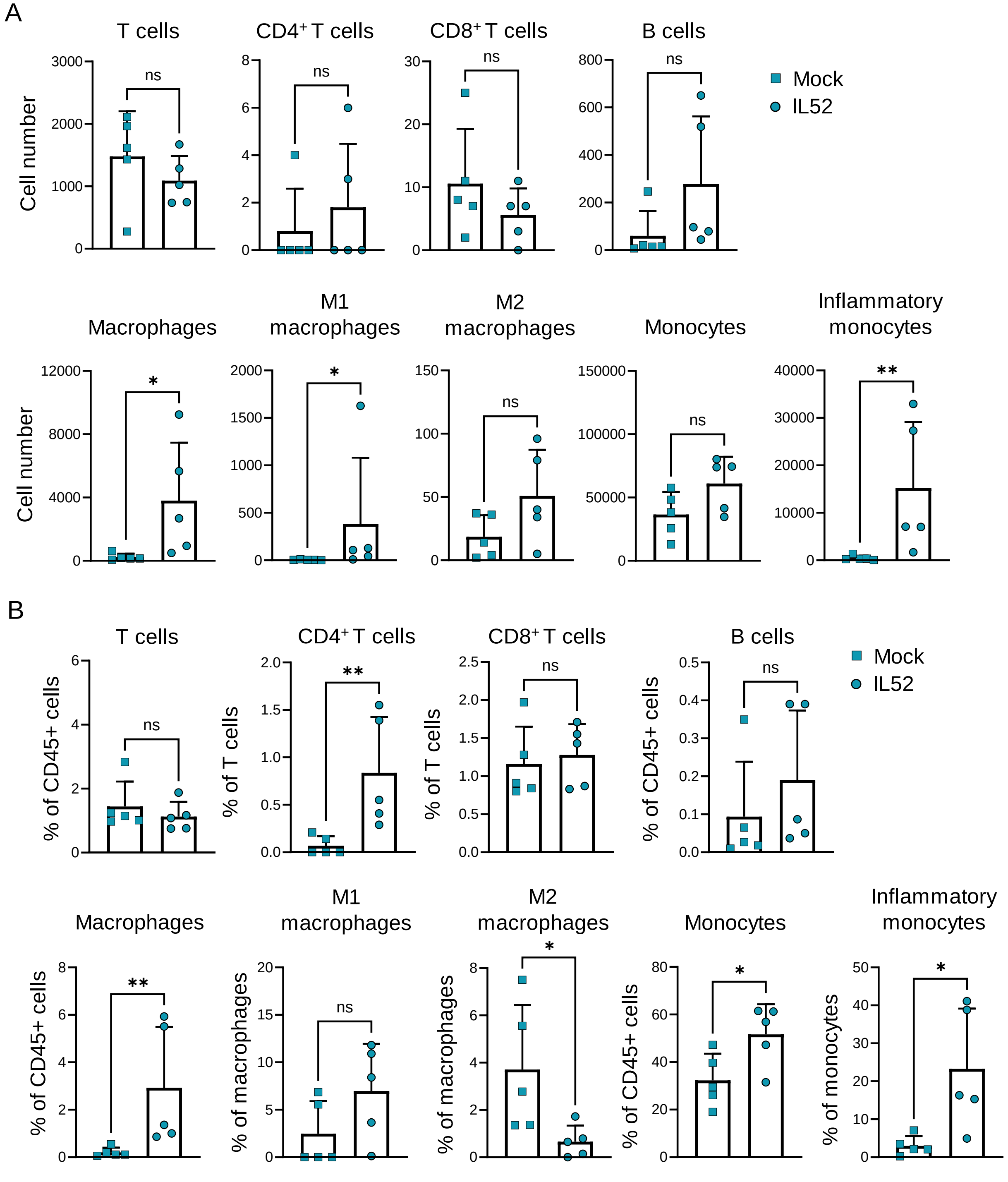

Supplemental Figure 7. *Rag1$^{-/-}$* mice have varied macrophage and T-cell populations following neurovirulent EV-D68 inoculation. Three-day-old *Rag1$^{-/-}$* mice were inoculated i.c. with PBS (mock) or EV-D68 IL52. Spinal cords were resected from paralyzed *Rag1$^{-/-}$* mice or day-matched mock-inoculated mice. Single-cell suspensions

were prepared, stained, and analyzed by flow cytometry. (**A**) Numbers and (**B**) percentages of selected cell types are shown. Data are representative of 2-3 independent experiments. Each symbol represents an individual mouse. Error bars indicate mean ± SD. Mann-Whitney test: *, $P \leq 0.05$; **, $P \leq 0.01$; ns = not significant.

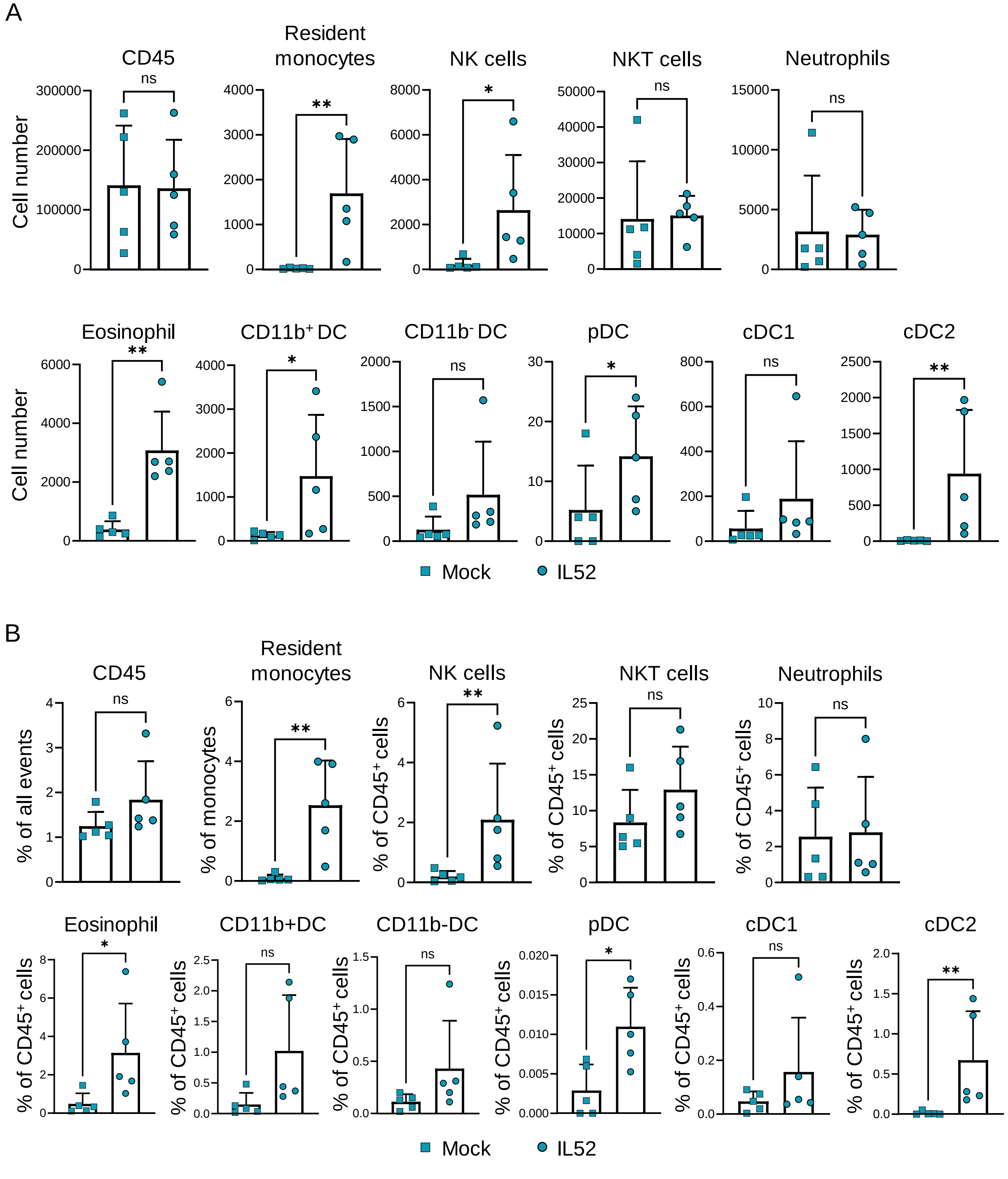

Supplemental Figure 8. Mice lacking mature lymphocytes have altered immune cell recruitment following neurovirulent EV-D68 inoculation. Three-day-old *Rag1*$^{-/-}$ mice were inoculated i.c. with EV-D68 IL52. Spinal cords were resected from paralyzed *Rag1*$^{-/-}$ mice or day-matched mock-inoculated mice. Single-cell suspensions were

prepared, stained, and analyzed by flow cytometry. (**A**) Numbers and (**B**) percentages of selected cell types are shown. Error bars are mean ± SD. Mann-Whitney test: *, $P \leq 0.05$; **, $P \leq 0.01$; ns = not significant. Each symbol represents an individual mouse. Data are representative of 2-3 independent experiments.
